# Cost-Effectiveness of Maintaining Higher Stem-Cell Collection Thresholds in the Chimeric Antigen Receptor T-Cell Era for Multiple Myeloma

**DOI:** 10.1200/CCI-25-00308

**Published:** 2026-03-20

**Authors:** Ehsan Malek, Brian Betts, Megan Herr, Marco Davila, Shernan Holtan, James J. Driscoll, Han Yu

**Affiliations:** ^1^ Roswell Park Comprehensive Cancer Center, Buffalo, NY; ^2^ University Hospitals, Seidman Cancer Center, Cleveland, OH; ^3^ Case Comprehensive Cancer Center, Cleveland, OH

## Abstract

**PURPOSE:**

Prolonged cytopenias are a common complication after chimeric antigen receptor (CAR) T-cell therapy for multiple myeloma, increasing the risk of severe infection. Infusion of previously collected autologous stem cells may mitigate this risk, but the clinical and economic implications of proactive collection remain uncertain.

**METHODS:**

We developed an 8-year, monthly cycle Markov model simulating 10,000 patients undergoing CAR T therapy. Two strategies were compared: (1) no stem-cell boost and (2) availability of a boost for patients with prolonged cytopenias. Transition probabilities for neutropenia, infection, and infection-related mortality were derived from CARTITUDE-4 and published stem-cell boost reports. Costs included hospitalizations for severe infection and upfront stem-cell reserve collection. Deterministic and probabilistic sensitivity analyses were performed.

**RESULTS:**

In the base case, universal reserve collection reduced severe infections from approximately 650 to approximately 260 per 10,000 patients and averted approximately 50 infection-related deaths. However, average per-patient costs were higher in the boost arm (approximately $19,700 US dollars [USD] *v* $4,500 USD), reflecting a gross reserve collection cost of $17,918 USD per patient plus lower residual infection-related hospitalization costs. Survival outcomes were similar between arms, with relapse-related mortality dominating long-term outcomes. Sensitivity analyses confirmed robustness, with hospitalization cost and reserve collection cost identified as the most influential parameters.

**CONCLUSION:**

Proactive stem-cell collection for CAR T recipients reduces infectious complications and modestly improves infection-related survival but remains economically unfavorable when applied universally. A risk-adapted approach targeting patients at highest risk of prolonged cytopenias may better balance clinical benefit with cost-effectiveness.

## INTRODUCTION

Chimeric antigen receptor (CAR) T-cell therapy has transformed the treatment paradigm for multiple myeloma (MM), providing a potent, one-time therapeutic strategy for patients with relapsed or refractory MM.^1^ This represents a major paradigm shift from the historical standard of continuous therapy that has long defined MM care. By targeting either B-cell maturation antigen (BCMA) or G protein-coupled receptor class 5 member D, CAR T-cell therapies have demonstrated remarkable response rates, often exceeding those of standard salvage regimens.^2,3^ However, their use is complicated by well-documented toxicities, mainly cytokine release syndrome (CRS), immune effector cell–associated neurotoxicity syndrome (ICANS), and immune cell–associated hematologic toxicity (ICAHT).^4^ These toxicities pose significant challenges in post-treatment management, affecting patient outcomes, health care utilization, and overall cost.

**CONTEXT**

**Key Objective**
Should transplant centers proactively collect additional stem cells for potential boosts in patients with multiple myeloma (MM) undergoing chimeric antigen receptor T-cell (CAR T) therapy?
**Knowledge Generated**
Using a Markov model informed by CARTITUDE-4 and published stem-cell boost data, universal collection reduced severe infections and infection-related deaths but substantially increased costs. Long-term survival remained dominated by relapse-related mortality.
**Relevance *(Z. Bakouny)***
Severe infections after CAR T-cell therapy in MM remain an unmet clinical need. The authors used a Markov model and found that universal collection of additional stem cells for potential boosts was not cost-effective, as reductions in infection-related costs did not offset increased collection expenses. Targeting this strategy to higher-risk patients may improve cost-effectiveness.*
*Relevance section written by *JCO CCI* Associate Editor Ziad Bakouny, MD, MSc.

Although CRS and ICANS tend to resolve within the first 2-week postinfusion period, ICAHT (ie, neutropenia, thrombocytopenia, or both) can persist for weeks to months, leading to sustained neutropenia and an increased risk of life-threatening infections.^5^

Prolonged ICAHT is characterized by cytopenia persisting beyond 30 days in a substantial proportion of patients.^6^ This prolonged bone marrow suppression has been attributed to multiple mechanisms, including direct myelotoxic effects of lymphodepleting chemotherapy, sustained inflammation, and disruption of the bone marrow microenvironment. ICAHT may correlate with heightened CAR T expansion and inflammatory milieu in some cohorts, although associations with treatment response remain inconsistent across studies.

Persistence of ICAHT not only increases the risk of infections and treatment-related morbidity but also prolongs dependence on transplant centers for ongoing supportive care. Patients with prolonged cytopenias often require close monitoring and frequent transfusions, which delays the transition of care to referring oncologists or community providers.

To mitigate ICAHT and accelerate hematopoietic recovery, autologous stem-cell boosts have emerged as a promising strategy. Existing stem-cell boost series most clearly suggest improvement in neutropenia-predominant ICAHT, while the optimal approach for thrombocytopenia-dominant cases remains uncertain and warrants further study. Infusing previously collected CD34^+^ stem cells can shorten the duration of neutropenia and reduce infection-related complications in patients with prolonged ICAHT.^7,8^ However, with the declining use of second autologous transplants,^9,10^ many centers have adjusted their stem-cell collection practices, often stopping once a minimum threshold adequate for a single autologous transplant has been achieved, hence limiting the availability of excess stem cells for future boosts. This shift raises a critical question regarding the optimal approach to stem-cell collection in the CAR T era. Should transplant centers proactively collect additional stem cells to support future boosts for CAR T patients, particularly those at high risk for ICAHT? This study evaluates the clinical and economic impact of extended stem-cell collection for future CAR T-cell therapy boosts using a Markov model framework.^11^ By comparing scenarios with and without stem-cell boosts, we aim to determine whether proactively collecting additional stem cells is a viable and beneficial strategy in the evolving MM treatment landscape, particularly in reducing long-term post-CAR T complications, health care resource utilization, and delays in transitioning care.

## METHODS

**FIG 1.**
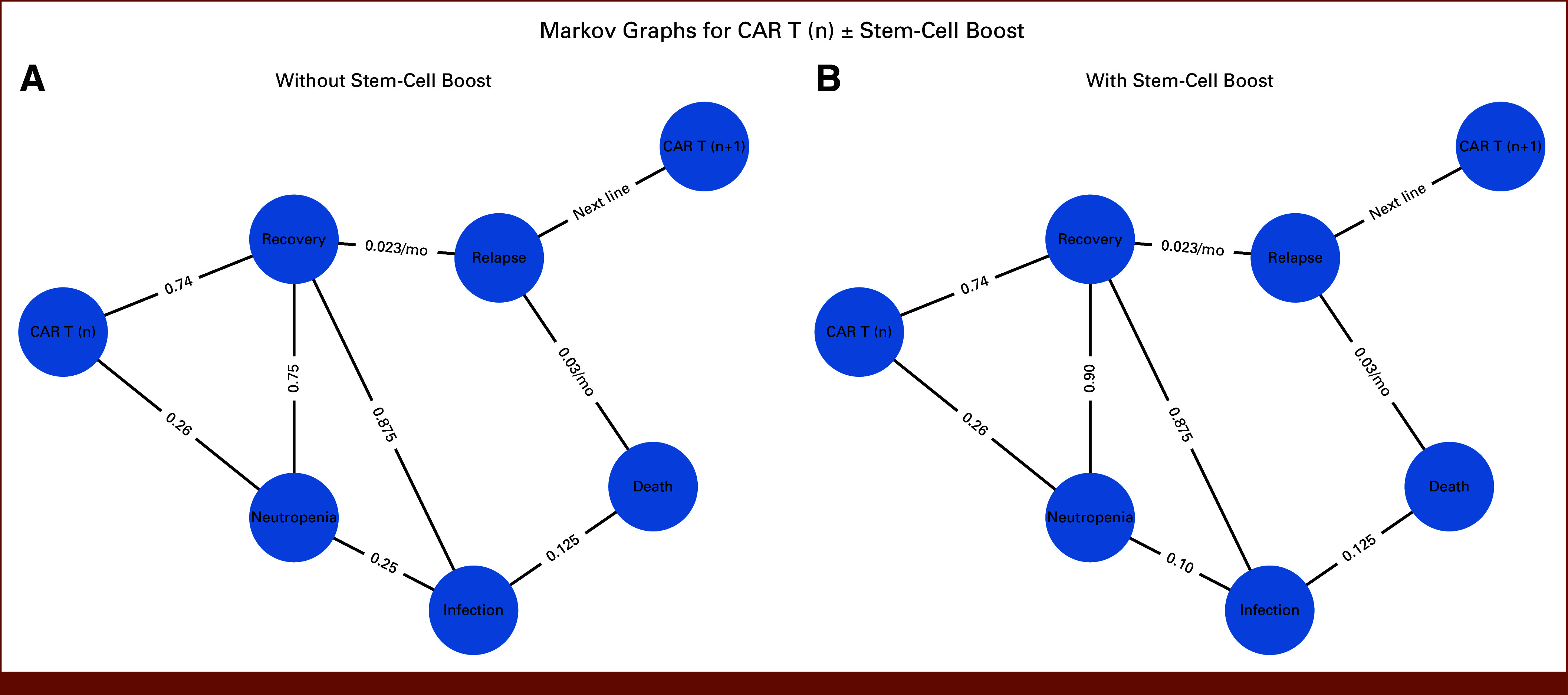
Markov model structure for patients undergoing CAR T-cell therapy (A) without or (B) with a planned stem-cell boost. States include CAR T (n), prolonged neutropenia (>30 days), infection, recovery, relapse, and death. In the illustrative schematic, a pathway from relapse to CAR T (n+1) is shown to reflect potential next-line therapy in clinical practice. This transition was not explicitly modeled in our base case but is included for conceptual completeness. Transition probabilities are shown on the arrows. In the no-boost arm, prolonged neutropenia leads to recovery in 75% or infection in 25% of patients; in the boost arm, probabilities are 90% and 10%, respectively. Infection carries a 12.5% case fatality, with survivors returning to recovery. Patients in recovery face a monthly relapse risk of 2.3% (95% CI, 1.7 to 3.0), based on PFS from the cilta-cel arm of CARTITUDE-4. Patients in relapse face a monthly relapse-related mortality risk of approximately 3% (calibrated to match CARTITUDE-4 OS). Death is modeled as an absorbing state. CAR T, chimeric antigen receptor T-cell; PFS, progression-free survival.

A Markov model was developed to simulate patient transitions across various health states over an 8-year period, using monthly time steps, resulting in 96 cycles. This framework is well suited for evaluating clinical strategies in settings where patients cycle through recurrent health states with different costs and outcomes, allowing estimation of cumulative benefits and resource utilization across large populations. The health states included CAR T-cell therapy, prolonged neutropenia lasting beyond 30 days, infection, recovery, relapse, and death. Two scenarios were analyzed: one in which patients received a stem-cell boost and another without the boost (Fig 1). The primary objective of the model was to quantify the financial impact of the stem-cell boost by evaluating total treatment costs and identifying key cost drivers. The Markov model was implemented in Python, with transition matrices constructed based on predefined probabilities derived from published clinical data. The model tracked the distribution of patients across different health states over time while calculating the total costs associated with each state. Sensitivity analysis and stress testing were automated to run multiple simulations with varying parameters.

**FIG 2.**
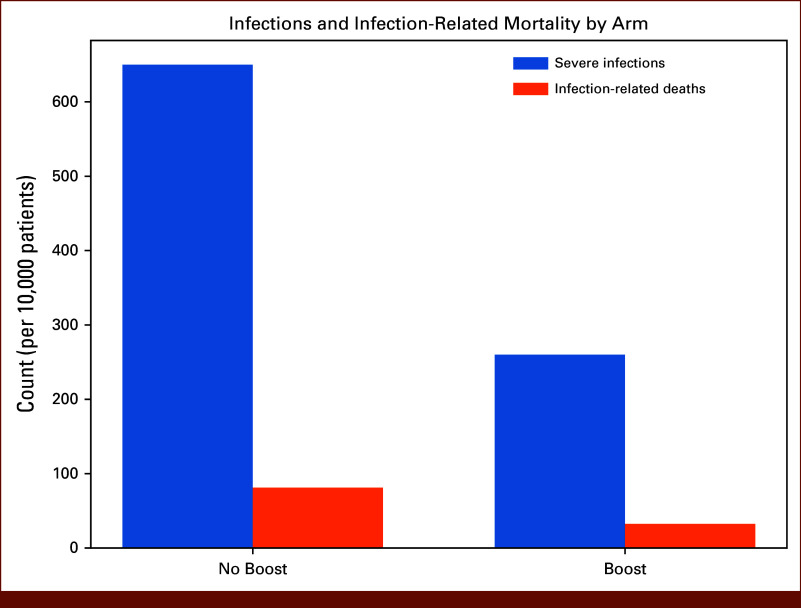
Infections and infection-related mortality by arm. A bar plot comparing the number of severe infections and infection-related deaths in the no-boost and boost strategies. The boost arm shows markedly fewer severe infections (n = 260 *v* 650) and averted infection-related deaths (50 fewer), reflecting the modeled 15% absolute reduction in infection risk.

**FIG 3.**
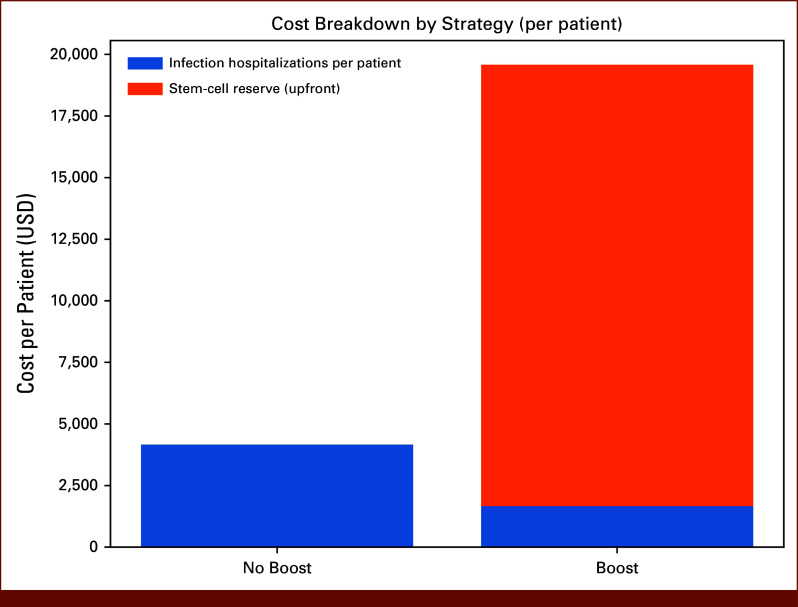
Cost breakdown by strategy. Stacked bar chart of per-patient costs for the no-boost and boost strategies. In the no-boost arm, all costs are attributable to hospitalization for infection. In the boost arm, upfront reserve collection accounts for the majority of costs, while infection-related costs are markedly lower. Total costs for each strategy represent the sum of upfront collection/storage fees and longitudinal infection-related hospitalization costs. USD, US dollars.

**FIG 4.**
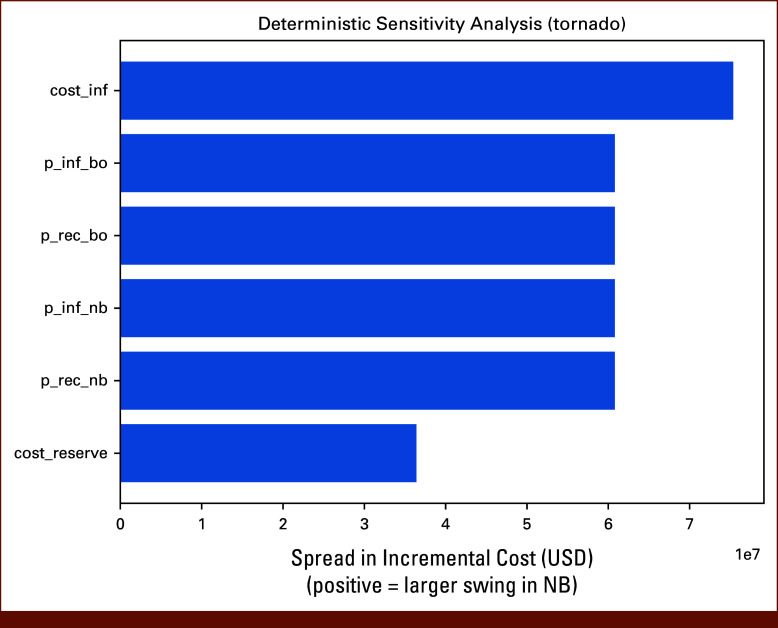
Deterministic sensitivity analysis (tornado diagram). One-way sensitivity analysis demonstrating the influence of key parameters on incremental cost. The cost of infection hospitalization and the reserve collection cost are the dominant drivers of uncertainty in incremental costs, while variation in neutropenia-to-infection probabilities had lesser impact. NB, no-boost; USD, US dollars.

**FIG 5.**
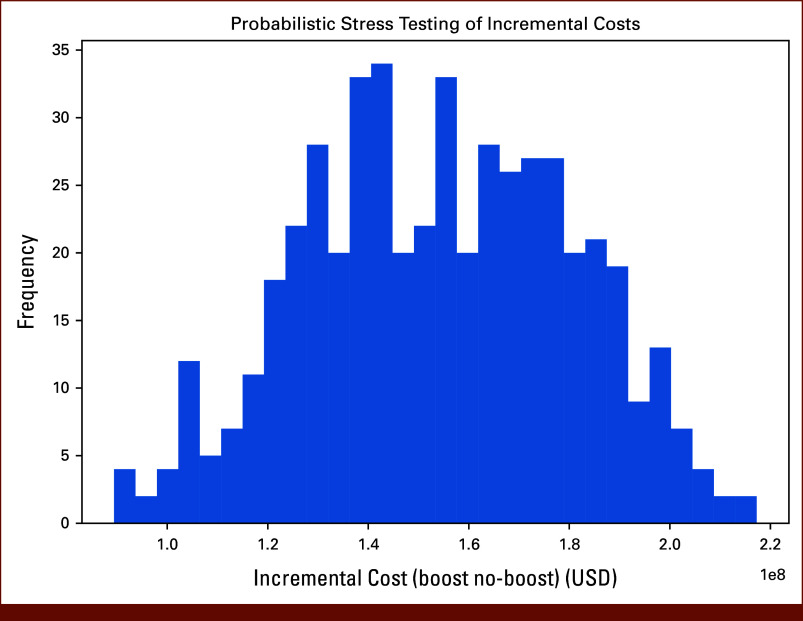
Probabilistic stress testing of incremental costs. Density histogram showing the distribution of incremental costs (no-boost minus boost) across 500 probabilistic draws. Nearly all simulations favored lower infection burden with the boost strategy, but the incremental cost remained positive in >95% of runs, confirming robustness of the base-case finding that universal boosting is more expensive. USD, US dollars.

### Transition Probabilities

The transition probabilities used in the model were derived from clinical trial data, particularly from the CARTITUDE-4 trial,^12^ and from the study by Mohan et al,^13^ which reported retrospective review of patients receiving a stem-cell boost to mitigate prolonged neutropenia after BCMA-directed CAR T-cell therapy. In this study, boosts were given selectively to patients with prolonged ICAHT, typically weeks after CAR T, using stored autologous grafts from previous mobilization. Since no patients in CARTITUDE-4 received a stem-cell boost, the probabilities for the *no-boost* scenario—including rates of prolonged neutropenia, infection, and infection-related mortality—were directly obtained from that data set. The *boost* scenario was parameterized using recovery and infection data from Mohan et al, where stem-cell infusion led to hematopoietic recovery in the majority of patients with persistent cytopenias. For the scenario without the stem-cell boost, the probability of transitioning from the CAR T state to prolonged neutropenia was set at 26%, reflecting the rate of grade 3 and 4 prolonged neutropenia in CARTITUDE-4. Among patients who developed neutropenia, the probability of recovery was set at 75%, while the probability of developing an infection was 25%. Although CARTITUDE-4 did not explicitly report the infection rate among those with prolonged neutropenia, these estimates were derived based on an overall infection rate of 62% in the CAR T arm, with 26.9% of infections classified as grade 3 or 4. To estimate the probability of death among patients with severe infections, data from CARTITUDE-4 were used to calculate that approximately 10%-15% of patients with prolonged grade 3-4 neutropenia would die from infection-related complications.

For the scenario incorporating a stem-cell boost, the probability of recovery from neutropenia was increased to 90%, while the probability of developing an infection was reduced to 10%. This adjustment was based on findings from Mohan et al, who reported significant hematopoietic recovery after a stem-cell boost. Specifically, the median absolute neutrophil count increased from 0.72 to 3.05 × 10^9^/L (*P* < .001), supporting the assumption that nearly 90% of patients receiving the boost would recover from neutropenia.^13^ The infection-related mortality probability remained unchanged at 10%-15%, as there was no direct evidence suggesting a significant alteration in infection-related mortality rates in the boost scenario.

Relapse was parameterized using progression-free survival (PFS) from the cilta-cel arm of CARTITUDE-4. The 12-month PFS was 75.9% (95% CI, 69.4 to 81.1). Under a constant hazard assumption, this translates to a monthly relapse probability of 2.3% (95% CI, 1.7% to 3.0%). Because both strategies in our model involve CAR T therapy, the same relapse process was applied to both arms. The probability of death while in relapse was calibrated so that model-predicted 12-month overall survival (OS) matched the observed 84% in the cilta-cel arm, which corresponded to a relapse-related monthly mortality of approximately 3%. Both relapse incidence and relapse-death probabilities were varied in deterministic and probabilistic sensitivity analyses to account for uncertainty in the constant-hazard assumption and the CI around observed PFS (Appendix 1).

### Model Implementation

The Markov model was implemented in Python, where transition probabilities were incorporated into transition matrices, governing the movement of simulated patients across health states over time. The simulation was conducted for 10,000 patients to ensure statistical reliability. The model was structured to accommodate both the boosted and nonboosted scenarios, allowing for a direct comparison of outcomes. Automated sensitivity analysis and stress testing were conducted to assess the robustness of the model under a range of parameter values. Since this analysis simulated patient transitions based on published transition probabilities rather than individual patient-level data, no baseline demographic or biologic characteristics (eg, age, sex, race, CHIP status, and previous treatments) are represented in the model. The simulated cohort reflects an aggregate risk profile consistent with clinical trial and real-world CAR T populations.

### Cost Assessments

Incremental cost was calculated as the difference between total per-patient costs in the boost and no-boost strategies. Hospitalization costs for severe infections were estimated using data from the study by Schilling et al, which examined inpatient costs for neutropenic complications in patients with hematologic malignancies.^13^ Adjusted for 2024 inflation, the average hospitalization cost for patients with neutropenia and infection was estimated at $64,012 US dollars (USD) per admission. The cost of collecting and storing additional stem cells for a future boost was estimated from the study by Ahmed et al,^14^ which analyzed expenses associated with peripheral blood stem-cell collection and storage for salvage transplantation in MM. The estimated cost, adjusted for 2025 inflation, was $17,918 USD per patient (Figs 2 and 3).

### Sensitivity Analysis and Key Driver Analysis

A sensitivity analysis was conducted to evaluate the robustness of the model by varying key parameters, including recovery probabilities, infection probabilities, hospital costs, and stem-cell boost costs. The transition probabilities for neutropenia recovery were tested at values of 0.70, 0.75, and 0.80, while the probability of infection was varied between 0.20, 0.25, and 0.30. For the boosted scenario, the probability of neutropenia recovery was tested at 0.85, 0.90, and 0.95, while the probability of infection was varied between 0.05, 0.10, and 0.15. The financial variables were also examined, with hospital costs ranging from $60,000 USD to $68,000 USD and boost-related costs ranging from $16,000 USD to $20,000 USD (Fig 4).

To identify the key cost drivers, a linear regression analysis was performed. Transition probabilities and cost variables were treated as independent variables, while cost savings, defined as the difference in total costs between the no-boost and boost scenarios, served as the dependent variable. The regression analysis provided insights into the relative contribution of each parameter to the overall financial impact of the stem-cell boost.

### Stress Testing

In addition to the sensitivity analysis, stress testing was performed to evaluate the model's robustness under extreme conditions. Recovery probabilities were tested within a broader range, from 0.60 to 0.85, while infection probabilities were varied between 0.15 and 0.35. These stress-test ranges (ST_REC_NB = 0.60-0.85; ST_INF_NB = 0.15-0.35) were selected to ensure logical consistency between recovery and infection probabilities. In the boosted scenario, recovery probabilities were tested at values ranging from 0.80 to 1.00, and infection probabilities ranged from 0.00 to 0.20. The financial inputs were also stress-tested, with hospital costs ranging from $50,000 USD to $70,000 USD and stem-cell boost costs ranging from $14,000 USD to $22,000 USD. The purpose of stress testing was to ensure that the model's predictions remained consistent even under highly favorable or adverse conditions (Fig 5).

## RESULTS

### Base-Case Analysis

In the base-case cohort of 10,000 patients followed over 8 years (96 monthly consecutive cycles), the model predicted a substantially lower burden of infection-related events with the availability of a stem-cell boost. In the no-boost arm, the model generated approximately 650 severe infections, whereas the boost arm experienced approximately 260 infections, reflecting the assumed 15% absolute reduction in infection risk (25% → 10% among patients with prolonged neutropenia). Infection-related mortality was reduced in parallel, with the boost strategy averting roughly 50 infection-related deaths compared with no-boost.

Relapse was modeled identically in both arms, with a monthly relapse probability of 2.3% and relapse-related mortality of 3% per month calibrated to cilta-cel OS. As expected, the relapse pathway dominated long-term mortality, while differences between arms were driven exclusively by the infection branch.

### Cost Outcomes

Total and per-patient costs differed between arms due to the upfront stem-cell reserve cost in the boost arm and fewer infection-related hospitalizations. The no-boost arm accrued an average per-patient cost of approximately $4,500 USD, reflecting infection-related hospitalizations only. By contrast, the boost arm incurred an average per-patient cost of approximately $19,700 USD, consisting of the $17,918 USD universal reserve collection cost plus residual infection–related hospitalization costs of approximately $1,800 USD per patient, reflecting the lower infection burden in this arm. Consequently, the incremental cost of the boost strategy was approximately $15,200 USD per patient over the modeled horizon. Cost breakdown analysis demonstrated that in the no-boost arm, nearly all costs were attributable to infection hospitalizations, whereas in the boost arm, the reserve cost dominated and infection costs were markedly lower.

### Sensitivity Analyses

Deterministic sensitivity analyses across the ranges specified in Methods (neutropenia recovery/infection splits and cost inputs) did not alter the direction of results. Across all combinations, the boost strategy consistently reduced the number of severe infections and infection-related deaths, but remained more costly due to the upfront reserve collection applied to all patients. The tornado analysis highlighted the cost of infection hospitalization and the reserve cost assumption as the most influential parameters on incremental cost. Stress testing with 500 probabilistic draws confirmed that >95% of simulations resulted in higher overall cost for the boost strategy, although the magnitude of incremental cost varied widely with hospitalization cost assumptions.

### Survival Outcomes

Because relapse probabilities and relapse-related mortality were modeled identically for both strategies, OS was nearly identical between arms. Differences in survival outcomes were limited to infection-related mortality, with the boost arm demonstrating a modest survival benefit from fewer infection deaths. Long-term survival remained dominated by relapse-related mortality in both strategies.

Although the model only captured hospitalization costs for severe infection and the upfront reserve cost, other health care resource utilization (eg, ICU stays, transfusions, and growth factor use) would likely further increase the clinical and economic rationale for stem-cell collection but were not explicitly modeled.

## DISCUSSION

The Markov modeling analysis reported here demonstrates that proactive collection of additional autologous stem cells for future boosts in patients undergoing CAR T-cell therapy meaningfully reduces the incidence of severe infections but comes at the expense of higher overall costs. This analysis focuses on the cost-effectiveness of maintaining higher stem-cell collection thresholds at the time of initial mobilization, ensuring adequate reserves for potential future boosts in the CAR T era. The boost strategy lowered both infection events and infection-related mortality, consistent with the clinical observation that stem-cell infusion can accelerate hematopoietic recovery in patients with prolonged cytopenias. However, because the reserve cost was applied universally in the boost arm, the incremental savings from reduced hospitalizations were insufficient to offset upfront collection and storage expenses.

Notably, a substantial subset of patients undergoing CAR T, particularly those who never underwent stem-cell mobilization or are older than 75 years, lack any previously collected reserves. These individuals represent the group most susceptible to prolonged hematologic toxicity and infection, highlighting the clinical importance of developing protocols that allow either early or contingency collection in appropriate candidates. Furthermore, with frontline BCMA CAR T trials underway (eg, CARTITUDE-5, ClinicalTrials.gov identifier: NCT04923893; KarMMa-4, ClinicalTrials.gov identifier: NCT04196491), ICAHT rates may decline in treatment-naïve patients who have not received high-dose melphalan. Although these shifts may alter future economics, autologous stem cell transplant remains standard in most settings, and access to early-line CAR T will remain limited in the near term. Accordingly, optimizing stem-cell collection at initial mobilization remains relevant during this transition period.

From a clinical perspective, the benefit of a stem-cell boost in mitigating infection-related complications represents an unmet clinical need. In our model, the reduction of approximately 390 severe infections and 50 infection-related deaths per 10,000 patients underscores the capacity of stem-cell infusion to lessen the morbidity and short-term mortality associated with immune cell–associated hematologic toxicity. Here, relapse transitions were parameterized using PFS data, which by definition captures both progression/relapse and death from any cause. This approach slightly overestimates relapse risk if nonprogression deaths occur with non-negligible frequency. However, in the CARTITUDE-4 trial, the gap between PFS and OS was modest in the first 12 months, suggesting that deaths without previous relapse were uncommon. Accordingly, we believe this approximation is reasonable for modeling purposes, although it should be recognized as a limitation. Nevertheless, long-term survival was dominated by relapse-related mortality, which was modeled identically in both arms based on PFS and OS data from CARTITUDE-4.

From an economic perspective, the dominant cost driver was the upfront reserve collection, which slightly outweighed the savings from fewer infection hospitalizations. Sensitivity and stress testing confirmed that this pattern persisted across a broad range of assumptions. The tornado analysis further emphasized that both the cost of hospitalization and the assumed reserve cost strongly influenced the incremental cost of the boost strategy. Importantly, our model only captured hospitalization costs for infection; other health care resources such as intensive care unit utilization, transfusions, or growth factor administration were not included in the cost-savings analysis. Incorporating these additional costs would likely increase the economic attractiveness of a boost, suggesting that our estimates may be conservative. Our model applied the cost of new stem-cell collection universally, which represents a conservative scenario. In practice, many patients already have stored cells from previous autologous transplant, and for these individuals, the boost strategy would likely be cost-effective because of substantially lower incremental costs.

By reducing severe infections and hospitalizations, the strategy should increase days at home, reduce transfusions/IV antibiotics, lessen caregiver burden, and speed up return to usual activities; in our base case, severe infections fell from approximately 650 to approximately 260 per 10,000 patients, and approximately 50 infection-related deaths were averted. The modeled OS gain is modest because relapse biology dominates long-term mortality, but the early reduction in infection deaths plus unmodeled quality-of-life benefits likely yield a favorable patient-centered impact. Prospective adoption should track the European Organisation for Research and Treatment of Cancer Quality of Life Questionnaire C30/Functional Assessment of Cancer Therapy-MM, days at home, unplanned readmissions, and treatment-continuity metrics, with potential for real-world OS to exceed modeled estimates if cytopenic windows shorten and care interruptions decline.

The results presented here have implications for transplant center practice. In settings where reserve collection costs can be minimized or where additional infection-related expenditures are substantial, the relative cost-effectiveness of stem-cell boosts may shift in favor of proactive universal collection. Conversely, in systems where reserve costs are high and supportive care costs are bundled, the economic rationale may be less compelling. Future studies should focus on identifying patient subgroups at highest risk of prolonged cytopenias, in whom the absolute reduction in infectious complications—and therefore the value of a boost—would be greatest. Importantly, the timing and coordination of stem-cell mobilization relative to CAR T collection remain open practical questions. Our model does not evaluate a same-cycle, just-in-time collection but rather assumes higher collection thresholds during initial autologous transplant mobilization. Nonetheless, centers exploring near-term collection strategies must balance risks of delayed bridging, chemomobilization requirements, and the theoretical concern of reinfusing tumor-contaminated cells. These logistical issues warrant prospective study to define feasible workflows and inform future cost-effectiveness modeling. In conclusion, proactive stem-cell collection in the CAR T era appears to provide clear clinical benefits in reducing infection risk and infection-related mortality but remains economically unfavorable when reserve costs are universally applied. These findings suggest that a risk-adapted approach, targeting collection to patients at highest likelihood of prolonged cytopenia using risk-stratification tools such as the CAR-HEMATOTOX score, may offer the most balanced strategy for optimizing both patient outcomes and health care resource use. Alternatively, BCMA-bispecific antibodies offer a cellular-sparing therapeutic option for patients at high risk for prolonged cytopenias or in whom extended stem-cell collection may not be feasible. As the treatment sequencing evolves, future decision frameworks may incorporate bispecifics as an alternative to autologous reserve collection in appropriately selected patients.

## Data Availability

A data sharing statement provided by the authors is available with this article at DOI https://doi.org/10.1200/CCI-25-00308.

## APPENDIX 1. DERIVATION OF RELAPSE PROBABILITIES

### Data Source

Relapse transitions were parameterized using progression-free survival (PFS) and overall survival (OS) data from the cilta-cel arm of the CARTITUDE-4 trial (N Engl J Med 2023; 389:335-47). The 12-month PFS was 75.9% (95% CI, 69.4 to 81.1), and the 12-month OS was approximately 84%.

Step 1: Conversion of PFS to a monthly relapse probability.

Under a constant hazard assumption, the cumulative hazard of progression at 12 months is:

H12 = –ln(S(12)), where S(12) is the 12-month PFS. The monthly hazard is then:

h = H12/12,

and the monthly probability of relapse (progression) is:

p_relapse, month = 1 – exp(–h).

With S(12) = 0.759, this yields:

H12 = 0.2758, h = 0.02298, p_relapse, month approximately equal to 0.0227 (2.27%).

Step 2: CI.

Applying the same transformation to the 95% CI for PFS (69.4 to 81.1) gives a monthly relapse probability range of approximately 1.7%-3.0%.

Step 3: Relapse-related mortality.

Patients in the relapse state face an additional monthly risk of death. This probability was calibrated so that the model-predicted 12-month OS matched the observed OS in CARTITUDE-4 (approximately equal to 84% for the cilta-cel arm), after accounting separately for infection-related deaths. In practice, this corresponded to a relapse-death probability of approximately 3% per month. Both relapse incidence and relapse-death transitions were varied in sensitivity analyses (±20%-30%) to reflect uncertainty.

Step 4: Sensitivity and probabilistic analysis.

1. Deterministic sensitivity: relapse incidence was varied by ±20% around the base-case 2.3%/month; relapse-death was varied by ±20%-30% around the 3%/month base-case.
2. Probabilistic sensitivity: relapse incidence was drawn from a log-normal distribution on the 12-month cumulative hazard. Specifically, if H12_hat = –ln(0.759), with bounds H12_lo = –ln(0.811) and H12_hi = –ln(0.694), then μ = ln(H12_hat) and σ = [ln(H12_hi)-ln(H12_lo)]/(2 × 1.96). For each probabilistic run, H12 approximately LogNormal(μ,σ^2^), and monthly relapse probability was set as one-exp(–H12/12). Relapse-death was sampled from a truncated normal distribution centered at 0.03 with standard deviation 0.005, bounded between 0.01 and 0.06.
3. Alternative specification: as a robustness check, piecewise hazards were generated from digitized PFS Kaplan-Meier curves using ht = –ln[S(t+1)/S(t)]. These produced qualitatively similar results to the constant-hazard assumption and did not change model directionality.

### Summary

The base-case monthly relapse probability was set at 2.3%/month (95% CI, 1.7 to 3.0), identical for both arms since all patients received chimeric antigen receptor T-cell therapy. A calibrated relapse-death probability of 3%/month ensured consistency with observed OS. Both parameters were varied extensively in sensitivity analyses.
